# Impaired left atrial reservoir and conduit strain in patients with atrial fibrillation and extensive left atrial fibrosis

**DOI:** 10.1186/s12968-021-00820-6

**Published:** 2021-11-11

**Authors:** Luuk H. G. A. Hopman, Mark J. Mulder, Anja M. van der Laan, Ahmet Demirkiran, Pranav Bhagirath, Albert C. van Rossum, Cornelis P. Allaart, Marco J. W. Götte

**Affiliations:** Department of Cardiology, Amsterdam UMC, Vrije Universiteit Amsterdam, Amsterdam Cardiovascular Sciences, De Boelelaan 1118, 1081 HV Amsterdam, The Netherlands

**Keywords:** Atrial remodeling, Atrial fibrillation, Atrial strain, Atrial fibrosis, Cardiovascular magnetic resonance (CMR)

## Abstract

**Background:**

Atrial fibrillation (AF) is associated with profound structural and functional changes in the atria. In the present study, we investigated the association between left atrial (LA) phasic function and the extent of LA fibrosis using advanced cardiovascular magnetic resonance (CMR) imaging techniques, including 3-dimensional (3D) late gadolinium enhancement (LGE) and feature tracking.

**Methods:**

Patients with paroxysmal and persistent AF (*n* = 105) underwent CMR in sinus rhythm. LA global reservoir strain, conduit strain and contractile strain were derived from cine CMR images using CMR feature tracking. The extent of LA fibrosis was assessed from 3D LGE images. Healthy subjects underwent CMR and served as controls (*n* = 19).

**Results:**

Significantly lower LA reservoir strain, conduit strain and contractile strain were found in AF patients, as compared to healthy controls (− 15.9 ± 3.8% vs. − 21.1 ± 3.6% *P* < 0.001, − 8.7 ± 2.7% vs. − 12.6 ± 2.5% *P* < 0.001 and − 7.2 ± 2.3% vs. − 8.6 ± 2.2% *P* = 0.02, respectively). Patients with a high degree of LA fibrosis (dichotomized by the median value) had lower reservoir strain and conduit strain compared to patients with a low degree of LA fibrosis (− 15.0 ± 3.9% vs. − 16.9 ± 3.3%, *P* = 0.02 and − 7.9 ± 2.7% vs. − 9.5 ± 2.6%, *P* = 0.01, respectively)*.* In contrast, no difference was found for LA contractile strain (− 7.1 ± 2.4% vs. − 7.4 ± 2.3%, *P* = 0.55).

**Conclusions:**

Impaired LA reservoir and conduit strain are present in AF patients with extensive atrial fibrosis. Future studies are needed to examine the biologic nature of this association and possible therapeutic implications.

**Supplementary Information:**

The online version contains supplementary material available at 10.1186/s12968-021-00820-6.

## Background

Atrial remodeling is an important hallmark feature of atrial fibrillation (AF) [1]. This process is characterized by inflammation, lipidosis, and fibrosis, leading to changes in atrial structure, geometry, volume, and function [2].

Cardiovascular magnetic resonance (CMR) imaging has emerged as the gold standard method to evaluate the atrial remodeling process. This imaging modality provides non-invasive assessment of left atrial (LA) wall fibrosis using three-dimensional (3D) late gadolinium enhancement (LGE) imaging [3, 4], as well as data on LA volume, geometry, and function. Assessment of LA function is complex, involving three phases, with important interplay between LA and left ventricular (LV) function in each phase. In systole, the LA serves as a reservoir, involving LA compliance, relaxation, and descent of LV base [5]. During early diastole, LA conduit function involves LV diastolic function (suction force) and LA compliance. At the end of diastole, LA contractile function depends on contractility, LV compliance, and end-diastolic pressure [6, 7]**.** CMR feature tracking post processing software has been developed for a more accurate assessment of atrial function in these three phases which can be used to determine the LA reservoir strain, conduit strain, and contractile strain [8, 9]. Recent studies have shown that LA fibrosis negatively impacts global atrial function [10, 11]. However, limited data exist on the relation between LA fibrosis and LA phasic strain. In the present study, we investigated the association between LA strain and LA fibrosis during all three phases of the cardiac cycle in AF patients, using advanced CMR imaging and post-processing techniques including 3D LGE and feature tracking.

## Methods

### Study design

This prospective single-center study was conducted in accordance with the Declaration of Helsinki. The study protocol was approved by the local medical ethics committee (Amsterdam UMC, location VU University Medical Center, Amsterdam, The Netherlands). Written informed consent was obtained from all individuals.

### Study population

A total of 105 AF patients were enrolled between July 2018 and April 2021. All patients had paroxysmal or persistent AF according to the European Society of Cardiology/EHRA guidelines [12, 13] and were scheduled to undergo a first pulmonary vein isolation (PVI) ablation procedure. Prior to the PVI catheter ablation, patients underwent CMR imaging while in sinus rhythm.

Exclusion criteria were general CMR contraindications (including metal implants and claustrophobia), contraindications for a gadolinium-based contrast agent, mechanical heart valves, a cardiac implantable electronic device, and absence of sinus rhythm as the time of CMR. Nineteen healthy subjects underwent CMR imaging without contrast administration to assess LA volume and function, and served as controls.

### CMR protocol

All scans were performed using a 1.5 T CMR (AVANTO or SOLA, Siemens Healthineers, Erlangen, Germany) and 32-channel array coil. The CMR protocol included balanced steady state free precession cine imaging in long axis orientations (two-chamber and four-chamber view), a 3D contrast enhanced magnetic resonance angiogram (CE-MRA) and 3D high resolution LGE images. For cine imaging, typical in-plane resolution was 1.3 × 1.3 mm^2^ and acquisition parameters were as follows: repetition time, 41–47 ms; echo time, 1.6 ms; slice thickness, 5 mm; flip angle, 60–75°; matrix, 256 × 208 mm; temporal resolution, < 40 ms.

An electrocardiogram (ECG) gated free-breathing navigator-based 3D CE-MRA of the LA and pulmonary veins was obtained immediately after a 20 mL (1 mL/sec) single dose bolus injection of contrast agent (Dotarem®, Guerbet LLC, Roissy, France) followed by a body weight dependent (0.2 mmol/kg) slow infusion of contrast agent (slow infusion dose; 2.5–30.0 mL, infusion rate; 0.1–0.25 mL/sec). A navigator (5 mm acceptance window) was positioned on the right hemi-diaphragm to acquire data during the end of respiratory expiration. Typical acquisition parameters were: repetition time/ echo time was 5.5/3.0 ms; flip angle, 25°; in-plane resolution was 1.25 × 1.25 mm with slice thickness 2.5 mm (reconstructed to 0.625 × 0.625 × 1.25 mm).

High resolution 3D LGE images were acquired using a navigator-based respiration- and ECG-gated inversion recovery prepared gradient echo pulse sequence applied approximately 20 min after contrast injection. The voxel size was 1.25 × 1.25 × 2.5 mm (reconstructed to 0.625 × 0.625 × 1.25 mm). Other typical sequence parameters were as follows: repetition time/ echo time was 5.2/2.4 ms; flip angle, 20°. Depending on the respiratory pattern and the heart rate of the patient, acquisition of the 3D CE-MRA and LGE series took approximately 10–15 min each.

### CMR data analysis

#### LA volume and function

Cine image analysis was performed using cvi42 (version 5.11, Circle Cardiovascular Imaging, Inc, Calgary, Alberta, Canada). Volumetric data of the LA and LV were calculated from two-chamber and four-chamber cine images using the biplane method. LA volume (LAV) was divided in minimal (LAV_min_), maximal (LAV_max_), and pre-atrial contraction volume (LAV_pre-c_). LAV_pre-c_ was defined as the last phase prior to atrial contraction. From these volumes, the total LA emptying fraction (LAEF) ((LAV_max_ − LAV_min_) × 100/LAV_max_), passive LAEF ((LAV_max_ − LAV_pre-c_) × 100/LAV_max_), and active LAEF ((LAV_pre-c_ − LAV_min_) × 100/LAV_pre-c_) were derived. LAV index maximum (LAVI_max_) was calculated by dividing LAV_max_ by body surface area.

#### LA strain assessment

Longitudinal LA strain analysis was performed using the feature tracking module in cvi42 (Circle Cardiovascular Imaging, Inc.). Endocardial and epicardial contours of the LA were traced in the end-diastolic phase of the long-axis two-chamber and four-chamber cine images. The automatic contour tracking algorithm was used and manual adjustments were applied, if necessary. This algorithm places a set of control points on the middle curve of the myocardial wall in the reference phase based on the drawn endocardial and epicardial contours. Subsequently, the position of the control points are detected based on the feature tracked boundaries in all the other phases to calculate longitudinal displacement.

**Fig. 1:**
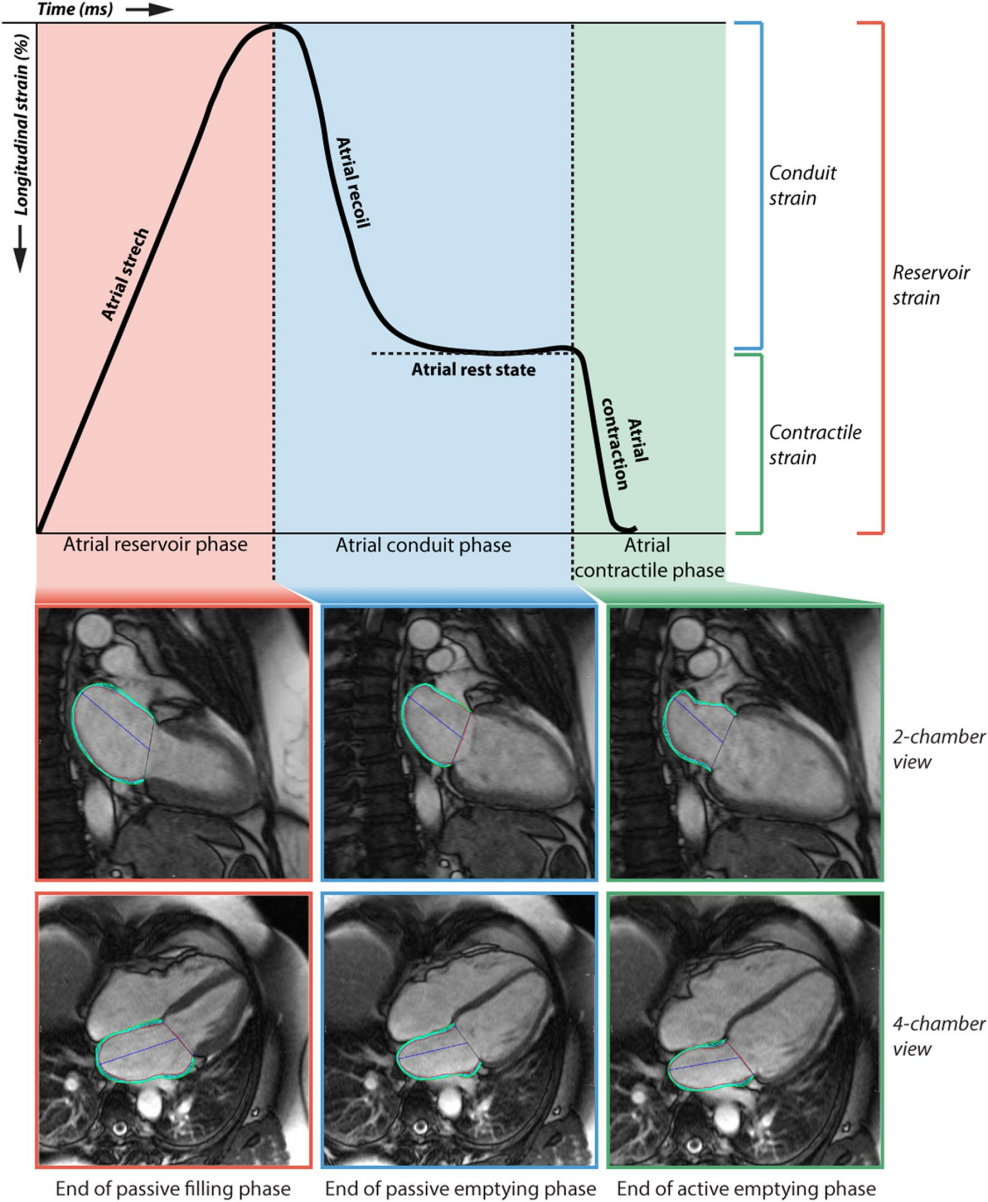
Left atrial (LA) strain during the 3 phases of the cardiac cycle. LA strain during the reservoir phase, conduit phase and contractile phase is illustrated, with corresponding two-chamber and four-chamber view, showing the LA endocardial and epicardial contours

Longitudinal strain measurements were subdivided into LA reservoir strain, conduit strain and contractile strain [14]. The time period (duration) for each phase was also calculated. Furthermore, LA peak positive strain rate, LA peak early negative strain rate, and LA peak late negative strain rate were measured using longitudinal strain rate curves. An illustration of LA strain analysis during the cardiac cycle is shown in Fig. 1 including a representative example of LA endocardial and epicardial contours.

#### Right atrial volume and strain assessment

Volumetric data of the right atrium (RA) were derived from the four-chamber cine images. RA volumes (RAV) (minimal (RAV_min_), maximal (RAV_max_), and pre-atrial contraction volume (RAV_pre-c_) as well as the total RA emptying fraction (RAEF), passive RAEF, and active RAEF were calculated. RAV index maximum (RAVI_max_) was calculated by dividing RAV_max_ by body surface area. Phasic RA longitudinal strain analysis was performed on the four-chamber cine images analogous to LA strain analysis as described above. A visual representation of the RA strain analysis is presented in the supplements (Additional file 1: Figure S1).

#### LA fibrosis and sphericity assessment

Quantification of LA fibrosis and calculation of LA sphericity was performed using open source software (CE-MRG (Cardiac Electro-Mechanics Research Group), King’s College London, United Kingdom) [15]. The LA blood pool including pulmonary vein extensions was segmented semi automatically in the 3D CE-MRA images on axial slices using a thresholding tool. The interpolated contours were adjusted manually in each axial plane. A 2 voxel (1.25 mm) surface dilation was used to define the epicardial border. Thereafter, the 3D CE-MRA images were co-registered with the 3D LGE images. A 3D reconstruction of the LA was generated and the LA appendage and the pulmonary veins were excluded at their ostia defined as the point of deflection from the LA wall. The mitral valve annulus was used to separate the LA from the LV. Signal intensity was normalized to the mean blood pool intensity according to the image intensity ratio method [16]. LA LGE was calculated using a default image intensity ratio threshold of 1.2 [17, 18], and was reported as percentage of the total LA surface.

In addition, the 3D LA shell obtained in CE-MRG was used to calculate LA sphericity using the algorithms published by Bisbal and colleagues [19]. In this regard, a LA sphericity of 100% represents a perfect sphere, whereas atria with a non-spherical shape will have a lower value.

### Reproducibility

Inter- and intraobserver variability for the LA strain analysis was assessed in 15 patients (by L.H. and A.D.). Reproducibility of LA LGE quantification was assessed in 15 patients (by L.H. and P.B.).

### Statistical analysis

Data are presented as mean ± standard deviation (SD) for normally distributed data and median including interquartile range (IQR) for data with a non-normal distribution. Normality of continuous data was assessed by inspection of histograms and Q-Q plots. To test for differences between two groups the Student *t*-test or Mann–Whitney *U* test was used, as appropriate. Pearson’s correlation was used to quantify associations between continuous variables. Intra- and inter-observer variability of LA strain measurements were assessed by intraclass correlation coefficients (ICC) for absolute agreement based on two-way random model. Data were considered significant if *P*-value < 0.05. Statistical analysis was performed using SPSS Statistics (version 26, Statistical Package for the Social Sciences, International Business Machines, Inc., Armonk, New York, USA).

## Results

### Patient characteristics

Good quality cine images were available in 90% of AF patients (94/105). Assessable 3D CE-MRA and 3D LGE images for quantification of LA fibrosis were available in 82 patients (78%). The baseline characteristics of the study population are presented in Table 1. In the AF group (*n* = 94), mean age was 60 ± 9 years and 64% were male. The study cohort consisted of 62 (66%) patients with paroxysmal AF and 32 (34%) patients with persistent AF. The median duration between AF diagnosis and CMR scan was 32 months (14–83 months). In the healthy control group (*n* = 19), mean age was 58 ± 4 years and 58% were male, which was comparable to the AF group.

**Table 1:** Baseline characteristics of the study population

	AF patients (n = 94)	Healthy controls (n = 19)	*P*-value
Demographics			
Age, years	60 ± 9	58 ± 4	0.21
Male gender	60 (64%)	11 (58%)	0.63
Weight (kg)	84 ± 14	80 ± 12	0.44
Height (cm)	180 ± 11	176 ± 8	0.15
BMI (kg/m^2^)	25.9 ± 3.4	25.6 ± 5.1	0.84
BSA (Mosteller)*	2.0 ± 0.2	2.0 ± 0.2	0.48
CHA_2_DS_2_-VASc score	1.2 ± 1.2	–	–
Hypertension	30 (32%)	–	–
Diabetes mellitus	4 (4%)	–	–
Medications			
ACE inhibitor or ARB	29 (30.9%)	–	–
Spironolactone	3 (3.2%)	–	–
Amiodarone	10 (11.0%)	–	–
Anticoagulation	77 (81.9%)	–	–
AF history			
Paroxysmal AF	62 (66%)	–	–
Persistent AF	32 (34%)	–	–
Time between AF diagnosis and CMR (months)	32 (14–83)	–	–

Values are expressed as number (percentage), mean ± SD or median (25–75th percentile). *ACE* angiotensin-converting-enzyme, *ARB* Angiotensin-receptor-blocker, *AF* atrial fibrillation, *BMI* body mass index, *BSA* body surface area, *CHA*_*2*_*DS*_*2*_*VASc* history of congestive heart failure, hypertension, diabetes mellitus, stroke/transient ischemic attack/prior thromboembolism, vascular disease, age and sex, *CMR* cardiovascular magnetic resonance. *Calculated by the Mosteller method ((height (cm) x weight (kg)/3600)^½^)

### LA volume in AF patients and controls

LA volumetric parameters are summarized in Table 2. LAVI_max_ was significantly higher in AF patients (49 ± 15 ml/m^2^ vs. 37 ± 8 ml/m^2^ in controls; *P* < 0.01). Also, a lower LAEF was observed in the AF patients (52 ± 13% vs. 64 ± 8% in healthy controls, *P* < 0.001).

**Table 2:** CMR characteristics of the study population

i	AF patients (n = 94)	Healthy Controls (n = 19)	*P*-value
LA volume			
LA volume—min (ml)	50 ± 28	26 ± 11	** < 0.001**
LA volume—max (ml)	100 ± 32	70 ± 15	** < 0.001**
LA volume index—max (ml/m^2^)	49 ± 15	37 ± 8	** < 0.01**
LA sphericity (%)*	79.5 ± 3.0		
LA function volumetric			
Total LAEF (%)	52 ± 13	64 ± 8	** < 0.001**
Passive LAEF (%)	27 ± 10	33 ± 8	** < 0.01**
Active LAEF (%)	25 ± 9	30 ± 7	**0.03**
LA strain			
LA reservoir strain (%)	− 15.9 ± 3.8	− 21.1 ± 3.6	** < 0.001**
LA conduit strain (%)	− 8.7 ± 2.7	− 12.6 ± 2.5	** < 0.001**
LA contractile strain (%)	− 7.2 ± 2.3	− 8.6 ± 2.2	**0.02**
LA peak positive strain rate	0.72 ± 0.24	0.87 ± 0.16	** < 0.01**
LA peak early negative strain rate	− 0.82 ± 0.33	− 1.22 ± 0.30	** < 0.001**
LA peak late negative strain rate	− 0.83 ± 0.30	− 0.97 ± 0.26	0.5
LA reservoir strain time (ms)	392 ± 45	383 ± 35	0.39
LA conduit strain time (ms)	430 ± 131	354 ± 101	**0.02**
LA contractile strain time (ms)	137 ± 41	123 ± 25	0.15
LA LGE (n = 82) (%)*	26.56 ± 16.0		
LV parameters			
LV ESV (ml)	69 ± 22	55 ± 12	** < 0.01**
LV EDV (ml)	168 ± 42	146 ± 27	**0.03**
LVEF (%)	59 ± 7	62 ± 5	0.08

Values are expressed as mean ± SD. *AF* atrial fibrillation, *bpm* beats per minute, *CMR* cardiovascular magnetic resonance imaging, *EDV* end diastolic volume, *EF* ejection fraction, *ESV* end systolic volume, *LA* left atrial; *LAEF* left atrial emptying fraction, *LGE* late gadolinium enhancement, *LV* left ventricular, *LVEF* left ventricular ejection fraction. Bold values denote statistical significance at the *P* < 0.05 level

*Not obtained in healthy volunteers due to the contrast agent dependency of the acquisition

### LA reservoir, conduit and contractile strain in AF patients and controls

**Fig. 2:**
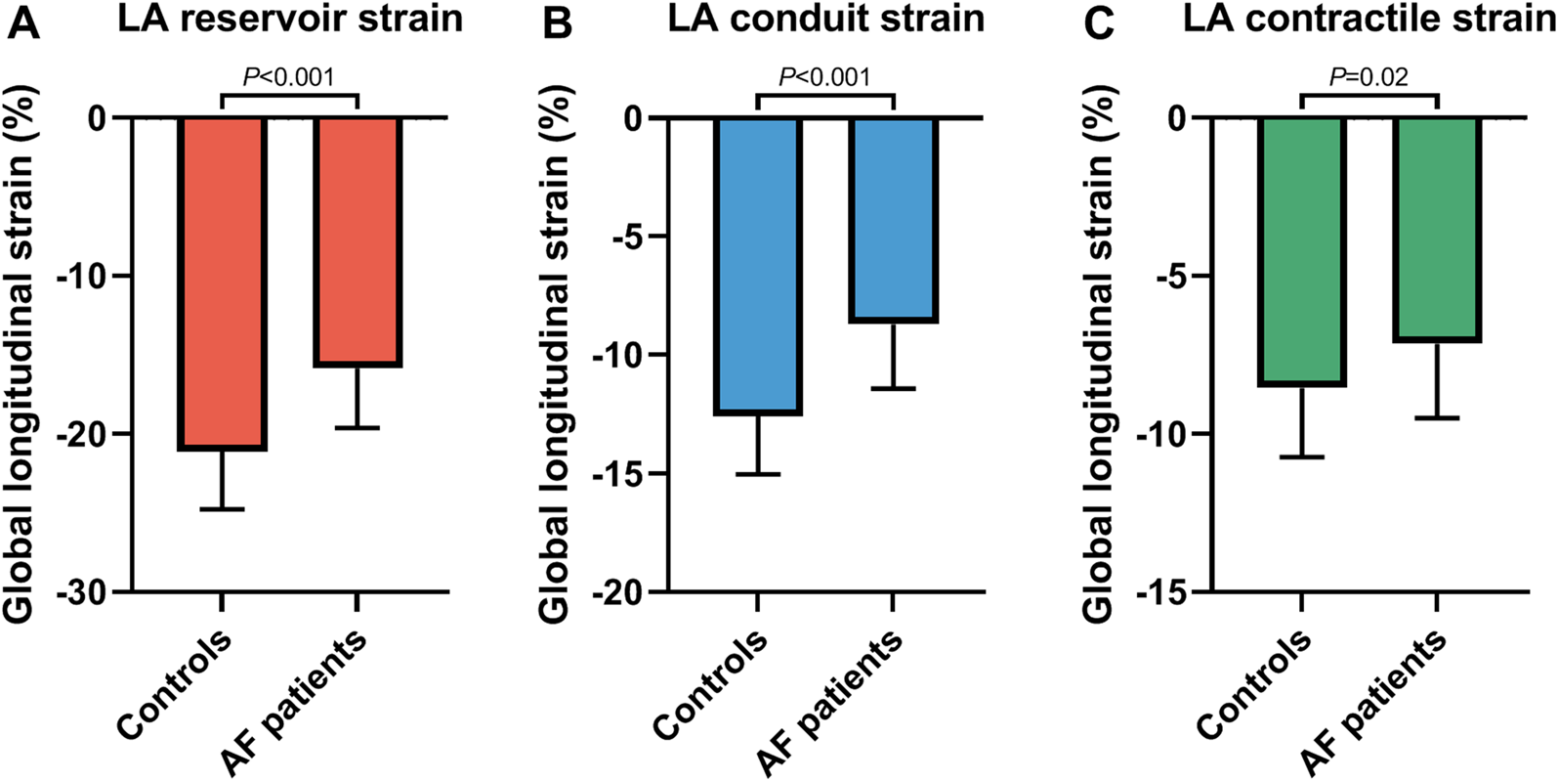
LA phasic strain in atrial fibrillation patients and healthy controls. **A** LA reservoir strain, **B** LA conduit strain and **C** LA contractile strain are impaired in patients with atrial fibrillation (AF), as compared to healthy controls. *AF* atrial fibrillation, *LA* left atrial. Data are presented as bars with mean and SD

In comparison with controls, all measures of phasic strain, i.e. reservoir strain, conduit strain, and contractile strain, were impaired in AF patients (− 15.9 ± 3.8% vs. − 21.1 ± 3.6% *P* < 0.001, − 8.7 ± 2.7% vs. − 12.6 ± 2.5% *P* < 0.001 and − 7.2 ± 2.3% vs. − 8.6 ± 2.2% *P* = 0.02, respectively; Fig. 2). LA conduit strain time was significantly different between AF patients (430 ± 131 ms vs. 354 ± 101 ms, *P* = 0.02) while LA reservoir strain time and LA contractile strain time were similar (Table 2). LA phasic strain was significantly associated with LAVI_max_ (reservoir strain; r = − 0.48, *P* < 0.001, conduit strain; r = − 0.33, *P* < 0.01, contractile strain; r = − 0.39, *P* < 0.001).

### RA remodeling as compared to LA remodeling in AF patients

Analysis of RA parameters could not be performed in 14 patients. RAVI_max_ was similar among AF patients and healthy controls (49 ± 16 ml/m^2^ vs. 45 ± 11 ml/m^2^, *P* = 0.52) while RAEF was different between these two groups (45 ± 10% vs. 52 ± 11%, *P* < 0.01, respectively). RA strain parameters were not different between AF patients and healthy controls (Additional file 1: Table S1). In AF patients, RAVI_max_ and LAVI_max_ had a weak but significant correlation (r = 0.33, *P* < 0.01) while RAEF and LAEF had no significant correlation (r = 0.21, *P* = 0.07). RA reservoir strain was correlated with LA reservoir strain (r = 0.36, *P* < 0.01) (Additional file 1: Figure S2).

### Impaired LA reservoir and conduit strain in AF patients with extensive fibrosis

**Fig. 3:**
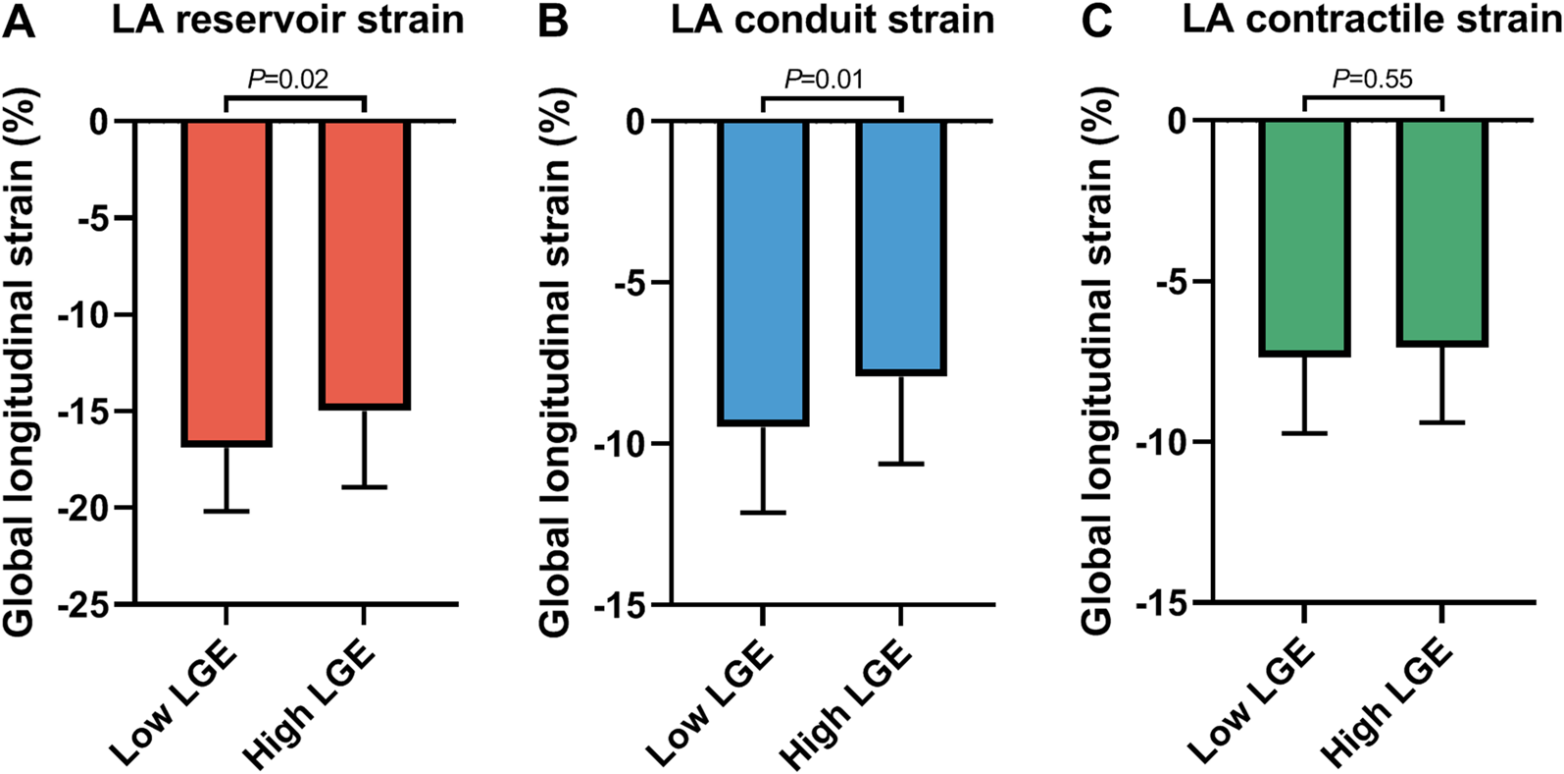
The association between the extent of LA late gadolinium enhancement and phasic LA strain in AF patients. **A** LA reservoir strain, **B** LA conduit strain and **C** LA contractile strain, stratified according to low and high degree of LA late gadolinium enhancement (LGE) in AF patients are depicted. *LA* left atrial, *LGE* late gadolinium enhancement. Data are presented as bars with mean and SD

To gain insight into the association between LA fibrosis and phasic strain, AF patients were dichotomized into groups according to the median percentage of LA LGE (low LGE ≤ 25.3% and high LGE > 25.3%) (Table 3). Age, LA volumes, and LA sphericity were comparable between AF patients with a low and high degree of LA LGE. Patients with hypertension were more often classified in the high degree LA LGE group (high LGE: 44% vs. low LGE: 22%, *P* = 0.03). Passive LAEF was significantly lower in patients with a high degree of LA LGE (24 ± 9% vs. 29 ± 11%, *P* = 0.03). Passive strain parameters, i.e. LA reservoir and conduit strain, were also significantly impaired in patients with a high degree of LA LGE (− 15.0 ± 3.9% vs. − 16.9 ± 3.3%, *P* = 0.02 and − 7.9 ± 2.7% vs. − 9.5 ± 2.6%, *P* = 0.01, respectively). Contractile strain however, was comparable between patients with high and low LGE (− 7.1 ± 2.3% vs. − 7.4 ± 2.4%, *P* = 0.55; Table 3, Fig. 3). LA conduit function showed the closest correlation to the extent of LA fibrosis (LA conduit strain Pearson correlation coefficient (r) =  − 0.33, *P* < 0.01; LA reservoir strain r =  − 0.29, *P* < 0.01; LA contractile strain r =  − 0.07, *P* = 0.53).

**Table 3:** Patient characteristics stratified according to low and high extent of LGE

	Low (≤25.3%) LGE (n = 41)	High (>25.3%) LGE (n = 41)	*P*-value
Age, years	59 ± 10	61 ± 7	0.42
BSA (Mosteller)*	2.1 ± 0.2	2.0 ± 0.2	0.15
CHA2DS2-VASc score	1.1 ± 1.2	1.3 ± 1.1	0.50
Hypertension	9 (22%)	18 (44%)	**0.03**
Diabetes mellitus	2 (5%)	2 (5%)	1.00
LA volume index—max (ml/m2)	48 ± 12	51 ± 16	0.36
LA sphericity (%)	79.7 ± 2.8	79.3 ± 3.3	0.61
LA function volumetric			
Total LAEF (%)	55 ± 11	50 ± 15	0.09
Passive LAEF (%)	29 ± 11	24 ± 9	**0.03**
Active LAEF (%)	25 ± 9	25 ± 11	0.98
LA strain			
LA reservoir strain (%)	− 16.9 ± 3.3	− 15.0 ± 3.9	**0.02**
LA conduit strain (%)	− 9.5 ± 2.6	− 7.9 ± 2.7	**0.01**
LA contractile strain (%)	− 7.4 ± 2.4	− 7.1 ± 2.3	0.55
LA peak positive strain rate	0.76 ± 0.27	0.70 ± 0.22	0.36
LA peak early negative strain rate	− 0.89 ± 0.31	− 0.76 ± 0.35	0.08
LA peak late negative strain rate	− 0.88 ± 0.28	− 0.80 ± 0.33	0.22
LA reservoir strain time (ms)	396 ± 51	383 ± 41	0.22
LA conduit strain time (ms)	452 ± 133	410 ± 130	0.15
LA contractile strain time (ms)	137 ± 35	132 ± 31	0.51

Values are expressed as number (percentage) or mean ± SD. *BMI* body mass index, *BSA* body surface area, *CHA2DS2VASc* history of congestive heart failure, hypertension, diabetes mellitus, stroke/transient ischemic attack/prior thromboembolism, vascular disease, age and sex, *LA* left atrium, *LAEF* left atrial emptying fraction, *LGE* late gadolinium enhancement. Bold values denote statistical significance at the *P* < 0.05 level

*Calculated by the Mosteller method ((height (cm) x weight (kg)/3600)^½^)

### Paroxysmal AF versus persistent AF

Finally, we compared parameters of LA and RA remodeling in patients with paroxysmal and persistent AF. Within the AF group, a higher maximal LAV and RAV was found in patients with persistent AF in comparison to patients with paroxysmal AF (LAV_max_: 113 ± 33 ml vs. 94 ± 30 ml, *P* = 0.02, RAV_max_: 110 ± 37 ml vs. 92 ± 32 ml, *P* = 0.03). No difference was found for LA sphericity (*P* = 0.52). Although LAEF and LA strain values tended to be lower in patients with persistent AF, no significant difference between the two groups was found. Finally, no difference was found in the extent of LA LGE between patients with paroxysmal AF and persistent AF (28.0 ± 13.8% and 23.9 ± 19.4%, *P* = 0.30) (Additional file 1: Table S2, Figure S3).

### Reproducibility

Fifteen randomly selected patients underwent repeated review to assess intra- and inter-observer reliability. The ICC for inter-reader variability of LA strain measurements was 0.85 (95% confidence interval: 0.73–0.91; L.H., A.D.). The ICC for intra-reader variability of LA strain measurements was 0.90 (95% confidence interval: 0.83–0.95). For LA LGE analysis, the ICC for inter-reader variability was 0.93 (95% confidence interval: 0.81–0.97; L.H., P.B.) and the ICC for intra-reader variability was 0.96 (95% confidence interval: 0.87–0.99).

## Discussion

Taking advantage of state-of-the-art CMR feature tracking to assess atrial strain, we demonstrated that all components of LA strain, i.e. LA reservoir, LA conduit, and LA contractile strain, are markedly depressed in AF patients as compared to healthy controls. A prolonged LA conduit strain time was found in AF patients compared to controls. Furthermore, we found lower LA reservoir strain and LA conduit in AF patients with extensive LA fibrosis. Interestingly, LA contractile strain was comparable between AF patients with low and high extent of fibrosis.

In the last decades, LA remodeling has been recognized as an important prognostic marker in AF [20]. AF induces a vicious circle of structural and functional remodeling that in turn instigates AF recurrence, which can ultimately become irreversible [21]. Timely interventions may decelerate and perhaps reverse this pathophysiologic process and improve clinical outcome. In this regard, detailed characterization of atrial remodeling, and understanding of the interplay between structural remodeling and function is essential.

It has been suggested that alterations in LA strain may precede the structural changes associated with LA remodeling [22, 23], making LA strain an interesting tool to monitor this process. Also, comprehensive assessment of all three functional phases of the LA may provide additional insight into LA remodeling, in addition to structural parameters such as LA volume, geometry and fibrosis solely.

In the present study, all components of LA strain i.e. LA reservoir, LA conduit and LA contractile strain, were impaired in the AF patients as compared to healthy controls, while all AF patients were in sinus rhythm during CMR. Also, a prolonged LA conduit strain time period was observed in AF patients which may be an expression of the enlarged and more ridged LA. The impaired phasic function was associated with a larger LA volume. Furthermore, a larger LA volume was found in patients with persistent AF, as compared to paroxysmal AF, which may be explained by a more advanced state of LA remodeling [10, 22]. Interestingly, there was no difference in LA volumetric function, LA strain, and LA fibrosis between patients with paroxysmal AF and persistent AF. A plausible explanation could be that persistent AF patients in our study cohort had either a mild form of persistent AF as all patients were in sinus rhythm during the scan. Moreover, the median time between AF diagnosis and the CMR scan was similar between patients with paroxysmal AF and persistent AF (paroxysmal AF: 35 months, persistent AF: 27 months, *P* = 0.75) suggesting that these patients may have a rather corresponding LA remodeling stage. Additionally, the clinical value of phasic LA strain in relation to conventional LA remodeling parameters for predicting recurrent AF after catheter ablation has yet to be established and hence will be studied in future research.

LA wall fibrosis is a hallmark of structural remodeling in patients with AF, and hence, might contribute to an impaired LA function [4, 17, 24]. Habibi et al*.* quantified LA fibrosis in AF patients and reported lower LA strain rates in the patients with a high degree of fibrosis [10]. Our results for the first time show lower LA reservoir strain and LA conduit strain in AF patients with more extensive LA fibrosis. Of interest, no association was found for contractile strain. Experimental studies have shown that atrial remodeling in AF is characterized by the presence of predominantly interstitial fibrosis and not by replacement fibrosis [25]. This interstitial fibrosis causes regional variation in myofiber architecture, which may impact atrial compliance, crucial for proper LA reservoir and LA conduit function [26, 27]. Likewise, interstitial fibrosis may impair LA reservoir and conduit strain, whereas active LA contractile function remains largely unaffected as cardiomyocytes are not replaced by fibrosis. This observation is supported in previous findings by Chesc et al. demonstrating a lack of association between active LAEF and the amount of LA fibrosis [28]. Structural alterations of the myocardium may have a greater effect on passive LA function while the LA contractile function may serve as a compensatory mechanism to maintain proper LV filling. As a results, contractile strain may not be different between patients with a high and low degree of LA fibrosis.

We found that AF is associated with structural and functional LA changes. Interestingly, minimal RA volume and RAEF were also different between healthy subjects and AF patients indicating a certain extent of RA involvement in AF. This bi-atrial remodeling was not characterized by a difference in RA strain between healthy subjects and AF patients. Literature on the relationship between RA remodeling and LA remodeling in AF patients is limited. A study by Xie et al. found that higher RA volume indices were independently associated with incident AF in a model adjusted for demographics and traditional risk factors while RAEF and RA global strain were not [29]. Moreover, the presence and impact of RA fibrosis detected by LGE-CMR in AF patients on RA function remains unknown and hence would be of interest for future studies.

### Limitations

Our study has several limitations. Firstly, there are multiple methods described for the detection of LA fibrosis using LGE-CMR [30]. In the present study, the IIR method was chosen to assess atrial fibrosis as this method is proposed to be a more consistent and reproducible method compared to algorithms relying on a certain number of standard deviations over a reference signal intensity. However, there is currently no complete agreement on IIR thresholds for LA LGE since direct histological validation on specific IIR cut-off values is lacking [31]. Based on recent literature, an IIR cut-off value of 1.2 was chosen to define atrial fibrosis [17]. Moreover, LA fibrosis quantification could not be performed in healthy subjects since the contrast agent based acquisition was not performed in this group. Therefore, no comparison could be made between the healthy control group and AF group in terms of LA LGE.

Secondly, the LA wall is thin (≈2 mm) and segmentation of the LA wall may be cumbersome in LGE-CMR images. Considering the thin atrial wall relative to the LGE-CMR voxel size, the CMR LA wall signal is subject to partial volume effects. Therefore, structures adjacent to the atrial wall such as the descending aorta may influence fibrosis quantification in this specific area. Moreover, the potential presence of inflammation and edema in the LA wall may have influenced the quantification of LA fibrosis using LGE [32, 33].

Thirdly, according to the EHRA AF guidelines, AF classification into either paroxysmal or persistent AF was based on *the more common AF type* the last six months. The patients classified as persistent AF patients in our study cohort were in sinus rhythm during the CMR scan and therefore may represent a selected category of persistent AF patients, having a mild form of persistent AF. This could explain the minimal differences in LA remodeling parameters between the two AF types. AF patients with ongoing persistent AF, often with a more advanced state of LA remodeling, were excluded from the study.

Lastly, because of LV/LA focused cine imaging, the RA could not be analyzed in a subset of patients. Moreover, RA volumes and strain were calculated only from the four-chamber cine images, while LA volumes and strain were calculated from two-chamber and four-chamber cine images using the biplane method. Assessment of the presence and impact of RA fibrosis in AF patients was not performed in this study and will be subject of future research.

## Conclusions

This study further establishes LA strain as marker of LA remodeling in AF patients, and for the first time demonstrates an important association between impaired (passive) LA reservoir strain and conduit strain in AF patients with extensive LA fibrosis. The amount of LA fibrosis, however, did not affect LA contractile strain. Future studies are required to study the biologic nature of this association and possible prognostic and therapeutic implications.

## Supplementary Information

**Additional file 1:** **Figure S1.** Right atrial feature tracking strain contours. **Figure S2.** Right atrial volumes and strain vs. left atrial volumes and strain in AF patients. **Figure S3.** LA volume and function in patients with paroxysmal and persistent AF. **Table S1.** Right atrial parameters of the study population. **Table S2.** CMR characteristics in patients with paroxysmal and persistent AF.

## Data Availability

The datasets used and/or analyzed during the current study are available from the corresponding author upon reasonable request.

## Abbreviations

3D: Three-dimensional
AF: Atrial fibrillation
CE-MRA: Contrast enhanced magnetic resonance angiography
CMR: Cardiovascular magnetic resonance
ECG: Electrocardiogram
EDV: End-diastolic volume
ESV: End-systolic volume
LA: Left atrium/left atrial
LAEF: Left atrial emptying fraction
LAV: Left atrial volume
LAVi: Left atrial volume index
LGE: Late gadolinium enhancement
LV: Left ventricle/left ventricular
LVEF: Left ventricular ejection fraction
PVI: Pulmonary vein isolation
RA: Right atrium/right atrial
RAEF: Right atrial emptying fraction
RAV: Right atrial volume
RAVi: Right atrial volume index
