# Supplementary material for: Impaired left atrial reservoir and conduit strain in patients with atrial fibrillation and extensive left atrial fibrosis
Source: J Cardiovasc Magn Reson. 2021 Nov 11;23:131. doi: 10.1186/s12968-021-00820-6 (PMC8582184; doi:10.1186/s12968-021-00820-6)
Supplement: Supplementary file 1 — Additional file 1: Figure S1. Right atrial feature tracking strain contours. Figure S2. Right atrial volumes and strain vs. left atrial volumes and strain in AF patients. Figure S3. LA volume and function in patients with paroxysmal and persistent AF. Table S1. Right atrial parameters of the study population. Table S2. CMR characteristics in patients with paroxysmal and persistent AF. [file 12968_2021_820_MOESM1_ESM.docx]

**ADDITIONAL FILE**

**Impaired left atrial reservoir and conduit strain in patients with atrial fibrillation and extensive left atrial fibrosis**

Luuk H.G.A. Hopman, Msc; Mark J. Mulder, MD; Anja M. van der Laan, MD, PhD; Ahmet Demirkiran, MD; Pranav Bhagirath, MD, PhD; Albert C. van Rossum, MD, PhD; Cornelis P. Allaart, MD, PhD; Marco J.W. Götte, MD, PhD

**Supplemental figures**

**Figure S1.** Right atrial feature tracking strain contours.

**Figure S2.** Right atrial volumes and strain vs. left atrial volumes and strain in AF patients.

**Figure S3.** LA volume and function in patients with paroxysmal and persistent AF.

**Supplemental tables**

**Table S1.** Right atrial parameters of the study population.

**Table S2.** CMR characteristics in patients with paroxysmal and persistent AF.

**Figure S1:** Right atrial feature tracking strain contours.


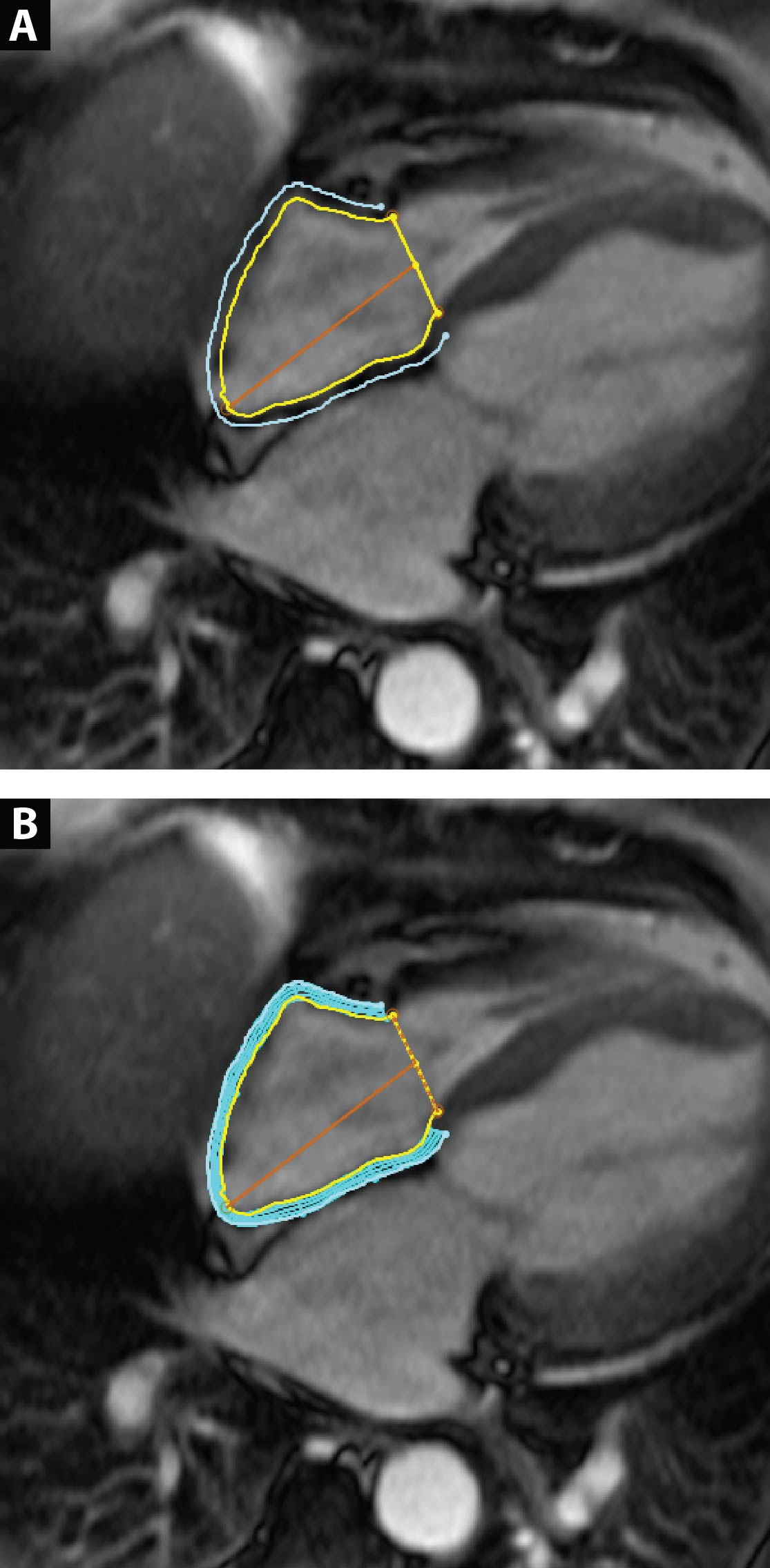


Cine four-chamber view showing (A) right atrial endocardial and epicardial contours and (B) right atrial contours with mesh overlay.


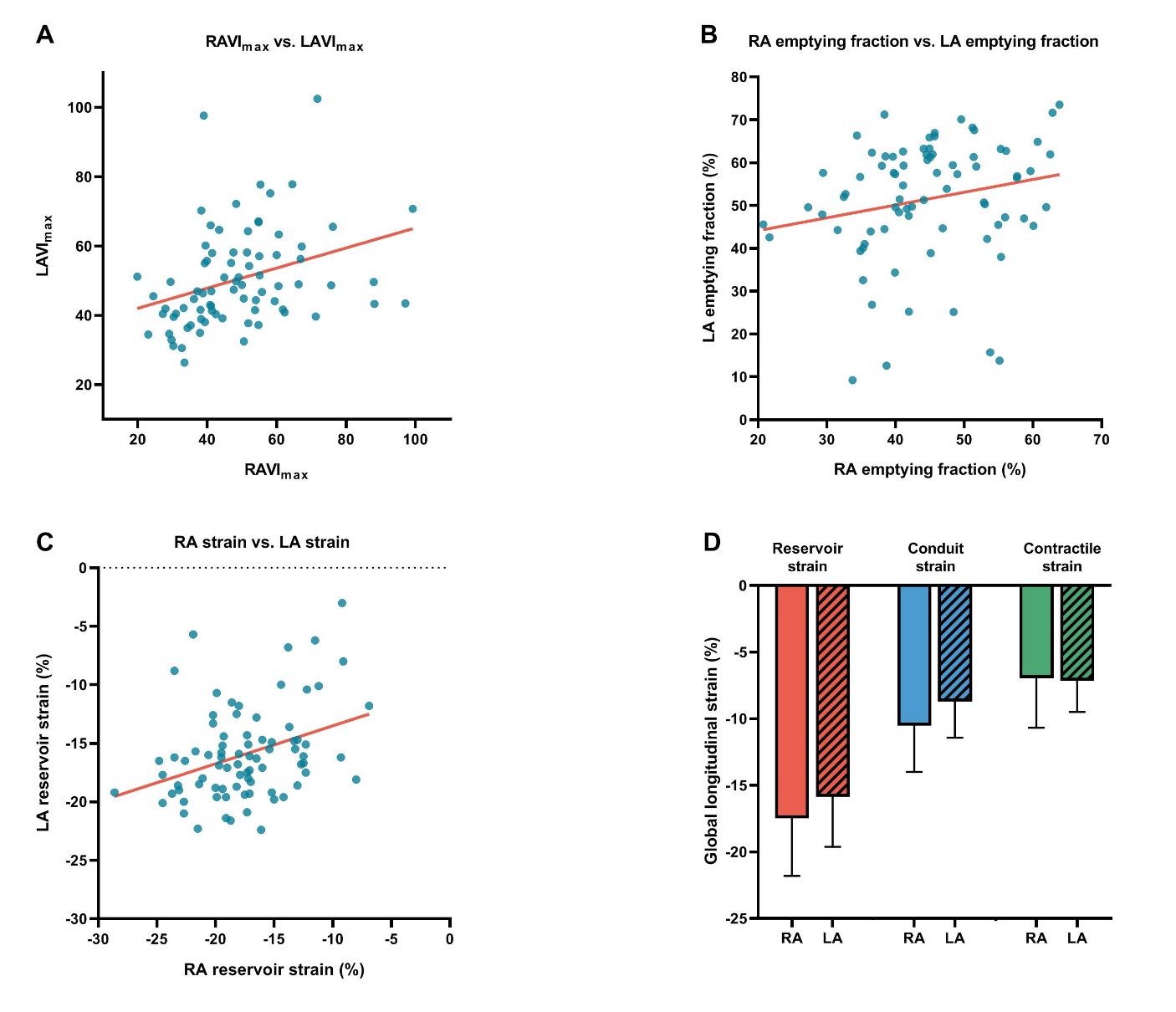
**Figure S2:** Right atrial volumes and strain vs. left atrial volumes and strain in AF patients.

(A) Scatterplot comparing maximal right atrial volume index and maximal left atrial volume index, (B) scatterplot comparing right atrial emptying fraction and left atrial emptying fraction, (C) scatterplot comparing right atrial reservoir strain and left atrial reservoir strain. (D) Bar-graph illustrating the right atrial and left atrial phasic strain values. Data are presented as bars with mean and SD.


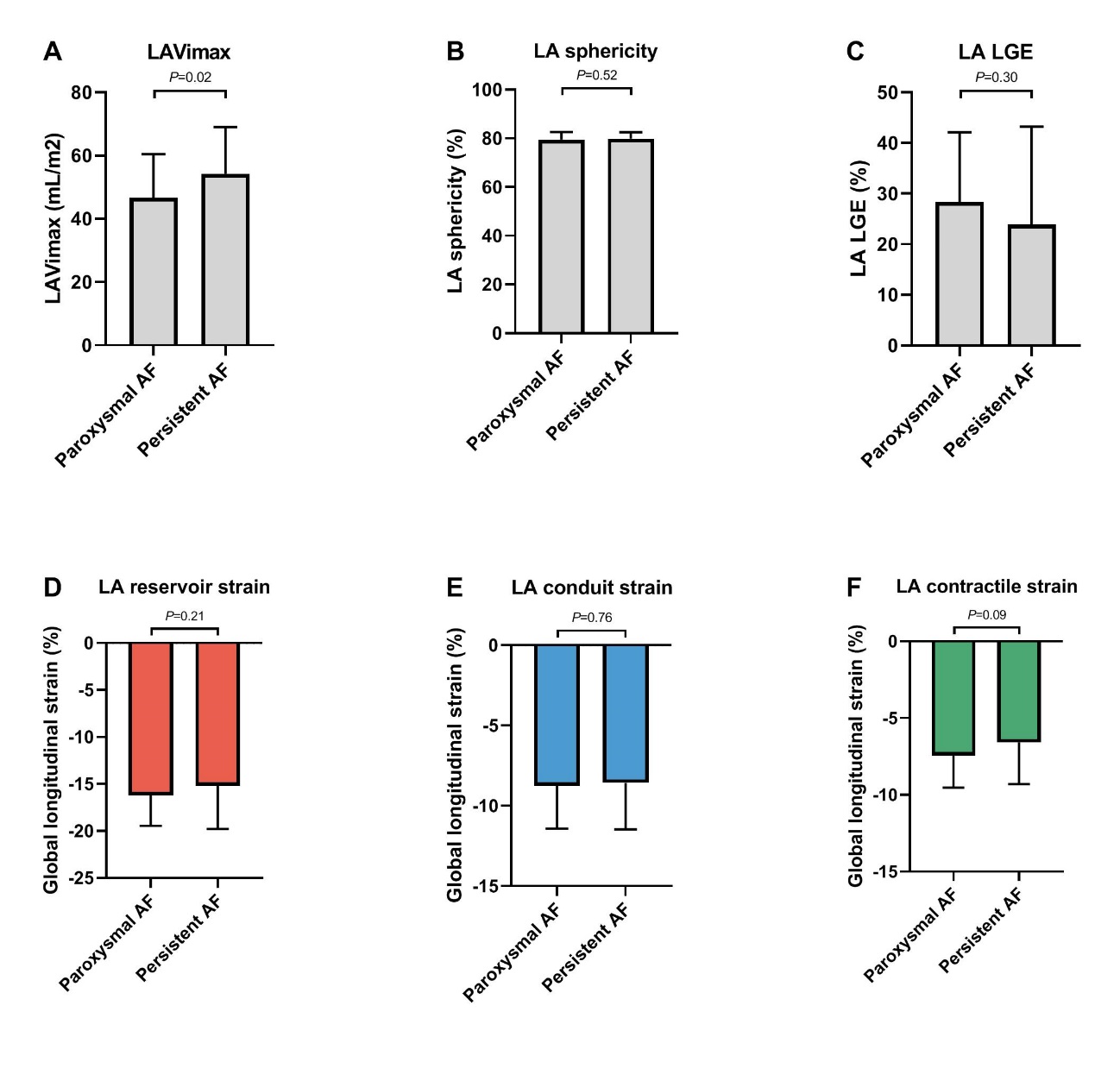
**Figure S3:** LA volume and function in patients with paroxysmal and persistent AF.

Differences in (A) LA LGE (%), (B) LAVi_max_ (mL/m^2^), (C) LA sphericity (%), (D) LA reservoir strain (%), (E) LA conduit strain (%) and (F) LA contractile strain (%) between patients with paroxysmal and persistent AF are depicted. AF, atrial fibrillation; LA, left atrial; LGE, late gadolinium enhancement; LAVi_max_, LA volume index – max. Data are presented as bars with mean and SD.

| **Table S1:** Right atrial parameters of the study population. | | | |
| --- | --- | --- | --- |
|  | **AF patients (*n*=80)** | **Controls (*n*=19)** | ***P*-value** |
| RA volume |  |  |  |
| RA volume - min (ml) | 55 ± 24 | 40 ± 14 | **0.001** |
| RA volume - max (ml) | 99 ± 35 | 84 ± 21 | 0.08 |
| RA volume index - max (ml/m^2^) | 49 ± 16 | 45 ± 11 | 0.52 |
| Total RAEF (%) | 45 ± 10 | 52 ± 11 | **<0.01** |
| RA strain |  |  |  |
| RA reservoir strain (%) | -17.5 ± 4.3 | -19.0 ± 3.2 | 0.16 |
| RA conduit strain (%) | -10.5 ± 3.5 | -10.9 ± 3.0 | 0.68 |
| RA contractile strain (%) | -7.0 ± 3.7 | -8.1 ± 2.9 | 0.22 |
| RA peak positive strain rate | 0.92 ± 0.29 | 0.90 ± 0.18 | 0.72 |
| RA peak early negative strain rate | -0.88 ± 0.31 | -0.83 ± 0.18 | 0.47 |
| RA peak late negative strain rate | -0.84 ± 0.31 | -0.85 ± 0.28 | 0.93 |
| RA reservoir strain time (ms) | 371 ± 44 | 339 ± 43 | **<0.01** |
| RA conduit strain time (ms) | 434 ± 143 | 382 ± 88 | **0.049** |
| RA contractile strain time (ms) | 143 ± 44 | 133 ± 34 | 0.22 |
| Values are expressed as mean ± SD. AF, atrial fibrillation; bpm, beats per minute; CMR, cardiovascular magnetic resonance imaging; RA, right atrial; RAEF, right atrial emptying fraction. Bold values denote statistical significance at the p < 0.05 level. | | | |

| **Table S2:** CMR characteristics in patients with paroxysmal and persistent AF. | | | |
| --- | --- | --- | --- |
|  | **Paroxysmal AF (*n*=62)** | **Persistent AF (*n*=32)** | ***P*-value** |
| Time between AF diagnosis and  CMR (months) | 35 (14 – 81) | 27 (16 – 85) | 0.75 |
| LA volume |  |  |  |
| LA volume - min (ml) | 45 ± 22 | 62 ± 36 | **0.02** |
| LA volume - max (ml) | 94 ± 30 | 113 ± 33 | **<0.01** |
| LA volume index – min (ml/m^2^) | 22 ± 11 | 29 ± 17 | **0.03** |
| LA volume index - max (ml/m^2^) | 47 ± 14 | 54 ± 15 | **0.02** |
| LA sphericity (%) | 79.4 ± 3.2 | 79.9 ± 2.6 | 0.52 |
| LA function volumetric |  |  |  |
| Total LAEF (%) | 54 ± 12 | 48 ± 16 | 0.09 |
| Passive LAEF (%) | 27 ± 10 | 25 ± 10 | 0.26 |
| Active LAEF (%) | 26 ± 9 | 23 ± 11 | 0.17 |
| LA strain |  |  |  |
| LA reservoir strain (%) | -16.2 ± 3.2 | -15.2 ± 4.6 | 0.21 |
| LA conduit strain (%) | -8.8 ± 2.7 | -8.6 ± 2.9 | 0.76 |
| LA contractile strain (%) | -7.5 ± 2.1 | -6.6 ± 2.7 | 0.09 |
| LA peak positive strain rate | 0.74 ± 0.21 | 0.67 ± 0.28 | 0.13 |
| LA peak early negative strain rate | -0.83 ± 0.34 | -0.78 ± 0.31 | 0.49 |
| LA peak late negative strain rate | -0.88 ± 0.29 | -0.73 ± 0.29 | **0.02** |
| LA reservoir strain time (ms) | 386 ± 44 | 404 ± 46 | 0.09 |
| LA conduit strain time (ms) | 429 ± 130 | 432 ± 135 | 0.92 |
| LA contractile strain time (ms) | 131 ± 45 | 149 ± 28 | **0.04** |
| LA LGE (%) (n=82) | 28.0 ± 13.8 | 23.9 ± 19.4 | 0.30 |
| RA volume |  |  |  |
| RA volume - min (ml) | 49 ± 21 | 66 ± 25 | **0.03** |
| RA volume - max (ml) | 92 ± 32 | 110 ± 37 | **0.03** |
| RA volume index – min (ml/m^2^) | 25 ± 10 | 31 ± 13 | **0.01** |
| RA volume index - max (ml/m^2^) | 46 ± 15 | 53 ± 18 | 0.09 |
| RA function volumetric |  |  |  |
| Total RAEF (%) | 47 ± 9 | 41 ± 9 | **<0.01** |
| Passive RAEF (%) | 23 ± 11 | 19 ± 8 | 0.10 |
| Active RAEF (%) | 24 ± 11 | 21 ± 10 | 0.31 |
| RA strain |  |  |  |
| RA reservoir strain (%) | -17.7 ± 4.8 | -17.1 ± 3.5 | 0.58 |
| RA conduit strain (%) | -10.5 ± 3.9 | -10.6 ± 2.8 | 0.94 |
| RA contractile strain (%) | -7.2 ±3.8 | -6.6 ± 3.7 | 0.47 |
| RA peak positive strain rate | 0.94 ± 0.26 | 0.88 ± 0.33 | 0.37 |
| RA peak early negative strain rate | -0.92 ± 0.34 | -0.82 ± 0.24 | 0.14 |
| RA peak late negative strain rate | -0.88 ± 0.34 | -0.77 ± 0.38 | 0.18 |
| RA reservoir strain time (ms) | 372 ± 41 | 370 ± 49 | 0.88 |
| RA conduit strain time (ms) | 420 ± 151 | 459 ± 126 | 0.24 |
| RA contractile strain time (ms) | 143 ± 46 | 143 ± 41 | 0.99 |
| LV parameters |  |  |  |
| LV ESV (ml) | 66 ± 18 | 74 ± 28 | 014 |
| LV EDV (ml) | 165 ± 37 | 174 ± 50 | 0.37 |
| LVEF (%) | 60 ± 7 | 57 ± 8 | 0.10 |
| Values are expressed as number (percentage) or mean ± SD. AF, atrial fibrillation; bpm, beats per minute; CMR, cardiovascular magnetic resonance imaging; EDV, end diastolic volume; EF, ejection fraction; ESV, end systolic volume; LA, left atrial; LAEF, left atrial emptying fraction; LGE, late gadolinium enhancement; LV, left ventricular; LVEF, left ventricular ejection fraction; RA, right atrial; RAEF; right atrial emptying fraction. Bold values denote statistical significance at the p < 0.05 level. | | | |
